# Notes of a Case of Diabetes Mellitus—Death and Autopsy—One Kidney and Ureter Only Discovered

**Published:** 1846-12

**Authors:** Geo. N. Burwell

**Affiliations:** Buffalo


ART. II.—*Notes of a Case of Diabetes Mellitus—Death and Autopsy—one Kidney and Ureter only discovered.* By GEO. N. BURWELL, M. D.

I was called, October 25th last, to visit O. D., aged 22 years. Irish born, but left Ireland in his infancy; lived in Canada until 1842, and in Buffalo since that year. Always healthy until the present sickness, and always strictly temperate.

Was taken suddenly with pain in his back one afternoon in July, 1843, after having been as well as usual all day, and having eaten a hearty dinner. This attack of pain was preceded by a sense of weakness, for which he had lain down in the shop where he was at work turning a lathe. The pain soon became so severe as to cause him to go home, and to bed. It continued all night. The next morning, for the first time, he had some difficulty and pain in urinating. He lay sick for six weeks or two months, with pain across his loins; some fever; general emaciation; urine scanty and passed with some difficulty, and depositing a brick-red sediment. He had then so far recovered as to get about, but still not strong enough to return to his work. He remained in this feeble condition until January, 1844, when he had an attack of the measles, from the effects of which he did not recover until February. In February he was much troubled with urticaria. This was followed by a cough, which lasted him all the spring and summer of 1844. He tried again to work, but had not sufficient strength: He therefore went to Canada, visiting, and walked a great deal in going from place to place.—One long walk in particular he remembers to have hurt him.

On his return to Buffalo in the Fall of 1844, he first had diabetes. This came on suddenly and simultaneously with anasarca, and with fever and thirst. Such has now been his condition for two years. None of the time has he been confined to his bed over a day or two at a time. He has generally been able to walk about. Neither the diabetes nor the anasarca has left him since they first appeared. Thinks he has from the commencement passed as much water as he does at present. Under medical treatment until the spring of 1846. He then went to the country to try the effects of living on a farm. Returned yesterday—having ridden more than fifty miles in an open lumber wagon, and over bad roads.

His principal complaint to-day is of pain in his left leg, about the cicatrix of an old ulcer. The leg is œdematous, and the skin reddened and inflamed—I fear ulceration. He says his bowels are regular; his appetite for hearty food voracious, and thirst great and uncontrollable. His tongue is of a deep red color, rough and pointed; skin very dry, and above the natural temperature; pulse 100, quick; face and legs œdematous. There was general emaciation, which was best seen on his arms, which were free from œdema; the sub-cutaneous fat was entirely removed. I directed his drink and urine to be measured for the next twenty-four hours.

Wednesday, Oct. 28. On visiting him I found he had drank twenty-four pints, and urinated about thirty pints, from 12 o'clock yesterday until 12 o'clock to-day. The urine was of a pale yellow color; acid to test paper; specific gravity 1,025; on adding nitric acid, a light purple-white haziness was at once produced; on allowing it to stand two or three hours there was a distinct deposit of albumen to be seen, the pink discoloration having disappeared. By heating the urine, an albuminous deposit was also produced, but less in amount than that obtained by nitric acid.

By testing it with nitric acid for urea, no approach to cystallization was produced. By the sulphuric acid test for sugar a carbonaceous deposit was formed; by adding yeast to it, there was rapid and large fermentation. His pulse had now risen to 120; he had great pain in his right chest, with difficulty of breathing; small white blisters had broken out all around his mouth; lips swelled and speech indistinct; restlessness great.

In the course of the afternoon the calls to urinate were much less frequent—moderate delirium set in, from which he could be so far roused as to recognise his friends; his pulse were to 140. He died on the morning of Oct. 29.

Oct. 30. Autopsy thirty-two hours after death. The general emaciation more marked than during life; œdema disappeared from the face and legs. For sufficient reasons the abdomen was the only cavity examined. There was large distension of the intestines by gas. The right kidney was first removed for examination. It was one-third to one-half larger than natural; flabby, although its substance was not softened; surface perfectly smooth. On making an incision lengthwise towards the fissure, more than the usual quantity of blood flowed from the cut. The general color of the kidney was a brownish tinge, both of the cortical and tubular portions. There was also another general but less marked tinge of yellow, arising from minute yellow dots all through the cortical substance of the kidney, but not prominent enough to give them the appearance of granulations. A number of the pyramids of Ferrien in different parts of the kidney had undergone a complete and entire yellow degeneration; the fibrous appearance yet remaining. A number of the malpighian bodies were noticed to be the centers of a much deeper slate color than the general tinge of the kidney. Most of these spots were circular, and from one-half to three-fourths of an inch in diameter. The pelvis of the kidney was healthy. The cortical substance was twice its natural thickness.

*The kidney and ureter of the left side were entirely wanting, no trace of them was to be found in any part of the abdomen.* The bladder was so distended as to occupy the whole of the pelvis and hypogastric regions, and probably contained from one to two quarts of water. Dr. Flint, the Editor of this Journal, was present at the autopsy, and, as well as myself, made a thorough examination for the left kidney and ureter.

*Buffalo, November*, 1846.

